# EEG Source Imaging: A Practical Review of the Analysis Steps

**DOI:** 10.3389/fneur.2019.00325

**Published:** 2019-04-04

**Authors:** Christoph M. Michel, Denis Brunet

**Affiliations:** ^1^Department of Basic Neurosciences, University of Geneva, Geneva, Switzerland; ^2^Center for Biomedical Imaging Lausanne-Geneva (CIBM), Geneva, Switzerland

**Keywords:** EEG, pre-processing, source localization, head model, inverse model

## Abstract

The electroencephalogram (EEG) is one of the oldest technologies to measure neuronal activity of the human brain. It has its undisputed value in clinical diagnosis, particularly (but not exclusively) in the identification of epilepsy and sleep disorders and in the evaluation of dysfunctions in sensory transmission pathways. With the advancement of digital technologies, the analysis of EEG has moved from pure visual inspection of amplitude and frequency modulations over time to a comprehensive exploration of the temporal and spatial characteristics of the recorded signals. Today, EEG is accepted as a powerful tool to capture brain function with the unique advantage of measuring neuronal processes in the time frame in which these processes occur, namely in the sub-second range. However, it is generally stated that EEG suffers from a poor spatial resolution that makes it difficult to infer to the location of the brain areas generating the neuronal activity measured on the scalp. This statement has challenged a whole community of biomedical engineers to offer solutions to localize more precisely and more reliably the generators of the EEG activity. High-density EEG systems combined with precise information of the head anatomy and sophisticated source localization algorithms now exist that convert the EEG to a true neuroimaging modality. With these tools in hand and with the fact that EEG still remains versatile, inexpensive and portable, electrical neuroimaging has become a widely used technology to study the functions of the pathological and healthy human brain. However, several steps are needed to pass from the recording of the EEG to 3-dimensional images of neuronal activity. This review explains these different steps and illustrates them in a comprehensive analysis pipeline integrated in a stand-alone freely available academic software: Cartool. The information about how the different steps are performed in Cartool is only meant as a suggestion. Other EEG source imaging software may apply similar or different approaches to the different steps.

## Introduction

The electric potential differences between electrodes placed on distinct scalp positions is due to the propagation of current flow induced by synchronized post-synaptic potentials of pyramidal neurons in the head according to Poisson's equations (1). However, this propagation is not homogenous. The current flow is strongly attenuated by the skull due to its high resistivity. This attenuation has to be properly modeled when solving the so-called forward problem, i.e., determining the potential at each scalp electrode generated by a known source in the brain (2). Since the thickness of the skull is not homogeneous across the head, it is highly recommended that the individual anatomical information derived from the MRI is used to determine the skull thickness and thus the local conductivity properties. Also, the shape of the head is not spherical and thus the distance of the electrodes to the center of the head is variable. The exact position of each electrode on the individual head should therefore be known. These properties (local skull thickness and 3D electrode position) are then incorporated in the lead field, which determines how the electric activity at a certain electrode is related to the activity of the different sources in the brain. The more precise and anatomically correct this lead field is determined, the more precise the source localization will be (3).

Once the proper head model has been built and the lead field is constructed, the second step consists in solving the inverse problem, i.e., determining the intracranial sources that generate a given EEG potential measurement on the scalp. This inverse problem is a fundamental challenge because a very large number of different source distributions can produce the same potential field on the scalp (4). Due to this non-uniqueness, a priori assumptions need to be incorporated (5). They can be purely mathematical or include neurophysiological, biophysical and anatomical knowledge about the distribution of neuronal activity in space and in time. It must be made very clear that no matter how sophisticated these assumptions and constraints are, the provided source solution remains an estimation that depends on how well-genuine sources conform to these assumptions (6). This holds for the EEG as well as for the MEG.

Localization of a limited number of equivalent dipoles is the most classical approach to solve the inverse problem (7). The a priori assumption in this solution is that only one or a few active areas in the brain generated the scalp potential field. Under this constraint, non-linear multidimensional optimization procedures allow to determine the dipole parameters that best explain the observed scalp potential measurements in a least-square sense (8, 9). The maximal number of dipoles that can be reliably localized depends on the number of scalp electrodes and is further limited by the non-linear complexity of the search algorithms with multiple sources (5). The number of dipoles can be increased by searching for the best solutions of dipoles with time-varying strength over a certain time period and by decoupling the linear and non-linear part of the estimation [BESA, (10), MUSIC, (11)]. It is important to be aware of the fact that if the number of dipoles is underestimated the source localization is biased by the missing dipoles. On the other hand, if too many dipoles are assumed, spurious sources will be introduced. Nevertheless, dipole source localization can produce reasonable results under some particular conditions (12), in particular in localizing the irritative zone in focal epilepsy (13–15) or the localization of primary sensory areas in evoked potentials, such as the sensorimotor cortex in surgical candidates (16). Dipole source localization is still widely used in the MEG community for these clinical applications (17).

Recent development in brain source imaging has offered more exciting options to localize brain sources from scalp EEG signals and have largely replaced the dipole source localization approach. These so-called distributed source localization methods do not make a priori assumption with respect to the number of dipoles. The most popular distributed source models currently used in the EEG community are modifications of a solution initially proposed by Hämäläinen and Ilmoniemi (18), called the Minimum Norm Solution (MN). The constraint introduced in this solution is that the current distribution over all solution points has minimum energy (minimizing the least-square error, i.e., the L2-norm) and that the forward solution of this distribution optimally explains the measured data. MN solutions are biased toward superficial sources because of their spatial vicinity to the sensors. Therefore, weighting parameters have been introduced to mitigate this bias, leading to the so-called weighted minimum norm (WMN) solutions (19–21). A variation of WMN is the low resolution electromagnetic tomography (LORETA) in which the norm of the second-order spatial derivative of the current source distribution is minimized to ensure spatial coherence and smoothness (22). This constraint has been justified by the physiological plausible assumption that activity in neighbored voxels are correlated. Another modification has been suggested by Grave de Peralta Menendez (23), called LAURA (Local AUtoRegressive Average). It incorporates the biophysical law that the strength of the source falls off with the inverse of the cubic distance for vector fields. LAURA integrates this law in terms of a local autoregressive average with coefficients depending on the distances between solution points. The general communality of all these linear inverse solutions is that they provide a distribution of the current density in the whole brain volume that is described as a 3D grid of discrete solution points. In each of these solution points, a current dipole with a certain orientation and strength is estimated. Usually, the space of these solution points is restricted to the gray matter (24). Several other linear and non-linear source localization algorithms have been described in the literature. This review focuses on the pre-processing steps that are needed for source localization and not on the characteristics of the different inverse solutions. For detailed discussions we refer to previous comprehensive review articles (3, 25–28).

In the following, we describe the different steps that are needed to get to these source localizations by illustrating them with the implementation in our freely available academic software package Cartool, a stand-alone program for the spatio-temporal analysis of EEG and evoked potentials (29), https://sites.google.com/site/cartoolcommunity/. The purpose of this concrete illustration is to explain in detail the points that are important to consider in each processing step and how they are implemented in Cartool. Several other powerful commercial or academic software packages for EEG source imaging exist that have implemented similar or alternative strategies. A comprehensive overview of different academic software applications can be found in a special issue of the Journal Computational Intelligence and Neuroscience (30), where programs such as BrainStorm (31), EEGLAB (32), FieldTrip (33), NUTMEG (34), SPM (35), and Cartool (29) are described. Widely used commercially available software packages for EEG/MEG source localization are BESA, Curry, GeoSource, and BrainVision Analyzer. Table 1 gives a summary of some of the most often used software packages and the source localization methods that they implemented. Whatever software is used, it is crucial that the user is aware and informed about the implementation of the different processing steps. In view of recent efforts to setup best practice guidelines of reporting EEG/MEG studies (https://cobidasmeeg.wordpress.com/), having access to the information of how the steps are done in the different software packages and reporting this information in the publications is important to ensure reproducibility and replicability.

**Table 1:** Non-exhaustive list of academic and commercial software packages that offer EEG source localization tools.

**Name**	**Website**	**Inverse models**
**ACADEMIC SOFTWARE PACKAGES**		
Brainstorm	https://neuroimage.usc.edu/brainstorm	Dipole modeling, Beamformer, sLORETA, dSPM
Cartool	https://sites.google.com/site/cartoolcommunity/	Minimum Norm, LORETA, LAURA, Epifocus
EEGLab	https://sccn.ucsd.edu/eeglab/index.php	Dipole modeling
Fieldtrip	http://www.fieldtriptoolbox.org/	Dipole modeling, Beamformer, Minimum Norm
LORETA	http://www.uzh.ch/keyinst/loreta.htm	LORETA, sLORETA, eLORETA
MNE	https://martinos.org/mne/stable/index.html	MNE, dSPM, sLORETA, eLORETA
NUTMEG	https://www.nitrc.org/projects/nutmeg	Beamformer
SPM	https://www.fil.ion.ucl.ac.uk/spm/	dSMP
**COMMERCIAL SOFTWARE PACKAGES**		
BESA	http://www.besa.de/products/besa-research/besa-research-overview/	Dipole modeling, RAP-MUSIC, LORETA, sLORETA, LAURA, SSLOFO
brainvision analyzer	https://www.brainproducts.com/	LORETA
BrainVoyager	https://www.brainvoyager.com/	Beamformer, Minimum Norm, LORETA, LAURA
GeoSource	https://www.usa.philips.com/healthcare/solutions/neuro/neuro-research-applications	Minimum Norm, LORETA, sLORETA, LAURA
CURRY	https://compumedicsneuroscan.com/curry-source-reconstruction/	Dipole modeling, MUSIC, Beamformer, Minimum norm, sLORETA, eLORETA, SWARM

## Basic Requirements

### EEG Pre-processing

Raw EEG data are contaminated by artifacts from many non-physiological (power line, bad electrode contact, broken electrodes, etc.) and physiological (cardiac pulse, muscle activity, sweating, movement, etc.) sources. These artifacts have to be carefully identified and either removed or excluded from further analysis. This is a cumbersome work and should be done by visual inspection of the raw data by experienced electrophysiologists. However, with the increasing availability of public EEG databases and the desire to analyze large datasets, the need for and the usage of automatic artifact detection and removal software is on the rise. Blindly applying such programs is problematic, because the type of artifacts is manifold and can vary in different experimental conditions. It is therefore recommended that if automatic artifact detection and correction methods are used, they should still be followed up by visual inspection of the data (36). In the following we describe the pre-processing pipeline implemented in Cartool.

### Temporal Filtering

Most studies first apply a temporal filter to the data in order to remove frequencies that are considered to be non-physiological and/or non-relevant for the study at hand. Since there is no consensus regarding the relevant frequency range and an increasing recognition of physiological relevance of frequencies below and above the conventional EEG frequencies [infraslow frequencies in resting state activity (37), high frequency oscillations in normal and pathological brains (38)], the range of the band-pass filter is driven by the study question. Resting-sate EEG is often filtered between 1–40 Hz, while evoked potential data usually considers broader frequency ranges (0.1–100 Hz). Filtering the data can have important effects on the time-courses and the phases of the data (39, 40), as well as on the localization of the waveforms' local extrema. This is of particular relevance in evoked potential studies, time-frequency analysis and connectivity measures. The exact characteristics of the filter that has been used should be described in the study report (36). In Cartool, we implemented a non-causal, Infinite Impulse Response (IIR) Butterworth filter of 2nd order, known for its optimally flat passband response, which limits the artificial introduction of new local maxima (41). Both Butterworth low- and high-pass filters have a−12 db/octave roll-off, and are computed linearly with forward and backward passes, which eliminates any phase shifts. This ensures that the local maxima will remain at their expected positions, irrespectively of their frequency content. In the specific case of Butterworth high-pass filtering, the D.C. value is explicitly removed beforehand, as very high baselines could cause IIR filters to become instable.

### Down-Sampling

After filtering, it is often useful to down-sample the data as most of the frequencies higher than the low-pass cutting frequency should be gone. It can dramatically reduce the memory requirements for the subsequent processing, without losing any information. The Nyquist theorem would require down-sampling not lower than twice the highest remaining frequency. In practice, though, because the filters' cut-offs are never perfectly sharp, and in order to keep some additional time resolution, the final sampling frequency should be chosen to be about four times the highest remaining frequency after low-pass filtering. In Cartool and for integer down-sampling ratios, down-sampling is done with a Cascaded Integrator-Comb (CIC) filter (42), which in practice is quite easy to compute in off-line applications. Other software packages, such as for example EEGLAB (32) apply antializing filters to reduce the sampling frequency.

### Electrode Interpolation and ICA

In Cartool, data inspection is performed semi-automatically. The user scrolls through the data and the program detects and visualizes electrodes with amplitudes above a certain range. If the user decides that a given electrode is an outlier due to bad contact, this electrode is marked and ignored in the subsequent independent component analysis (ICA).

The ICA is used to detect and correct artifacts, particular eye movements, eye blinks and cardiac pulse artifacts (43). It is important that the time course of the ICA components that are considered to reflect one of these artifacts is inspected together with the raw EEG data and it is assured that they indeed spatially (topography) and temporally correlate with the appearance of these events. Once this is assured, the data are back-projected by excluding these components. At the time of publication, ICA is not fully implemented in Cartool. An often used software for artifact removal using ICA is EEGLAB (44).

After ICA correction, the bad electrodes detected in the first step are interpolated using a 3D or spherical spline algorithm (45). In order to do that, the 3-dimensional position of each electrode needs to be known (see section Determining the Solution Points in the Gray Matter.).

### Spatial Filtering

The precursor of EEG source imaging is the scalp potential map (5). Therefore, visualizing and inspecting the quality of the topography of the maps is as important as the inspection of the waveforms. Even after interpolation of artifacted electrodes and removing irrelevant ICA components, transient events can corrupt a few electrodes for a short time period. They can be seen on the potential map displays as isolated “islands” within the local neighborhood. Such outlier electrodes will have dramatic effects on source localization as the steep gradients will lead to local maxima beneath the electrode [see Figure 4.7. in (46)].

Here we describe a spatial filter that we designed and implemented in Cartool. It is an instantaneous filter which removes local outliers by spatially smoothing the maps without losing its topographical characteristics.

**Figure 1:**
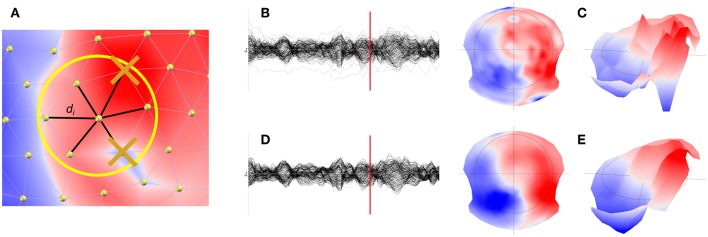
Illustration of the spatial filter implemented in Cartool. **(A)** Determination and removal of the maximal and minimal value of the 6 nearest neighbor of a given electrode. **(B)** Illustration of the waveforms and the map **(C)** at a given time point before filtering. **(D)** Illustration of the effect of the spatial filter on the waveforms and maps **(E)**.

The spatial filter is designed in the following way (see Figure 1A):

- For each electrode, the values of the 6 closest neighbors are determined, plus the central electrode value itself.
- The 7 data points are sorted.
- The minimal and maximal values are removed by dropping the first and last items of this list.
- The remaining 5 values are then averaged, with weights proportional to the inverse distance to the central electrode. The central electrode is given a weight of 1.

This is very similar to an Interquartile Mean (IQM), but cutting the Cumulated Density Function into 7 intervals instead of 4, so we technically have an Inter Septile Weighted Mean. For each electrode *e*:

(1) $$SpatialFilter\left(e\right)=\left(\sum_{i=1}^{i=5}\frac{v_{i}}{d_{i}}\right)/\left(\sum_{i=1}^{i=5}\frac{1}{d_{i}}\right)$$

With *v*_*i*_ being the 5 remaining voltage values from the 6 nearest neighbors of electrode *e*, plus the central value, each being at distance *d*_*i*_. An example of the effect of the spatial filter on the waveforms (Figures 1B,C), but more importantly on the topography, can be seen in Figures 1D,E.

### Detecting Bad Epochs

Hopefully, at this stage the EEG data is clean enough for further processing. Still, transient artifacts may remain (muscle artifacts, sweating, remaining eye blinks, etc.) that none of the steps above successfully removed. It is therefore strongly recommended that the “cleaned” data are visually inspected and that bad epochs are marked. In Cartool, we have implemented a tool that helps to identify these bad epochs. It is based on a set of simple statistics on the tracks and then estimates how much each track deviates from its own individual baseline. The statistics is based on instantaneous values (absolute value, variance, skewness and kurtosis among electrodes at a given time point) and on short time periods by computing the cross-convolution, which is a convenient way to estimate the noise in a signal. All these outlier estimators are merged together to a single compound estimator and the highly suspicious time periods are highlighted. By visual inspection, the user can then decide whether these periods should be marked as “bad” or not. These bad epochs will be conveniently used in later processing, as many toolboxes of Cartool allow to skip them.

## Constructing the Head Model

The head model is the model for which the EEG forward solution is calculated. The forward solution determines how much a given electrical source in the brain will impact each electrode on the scalp. It provides the lead field matrix from which the inverse problem will be solved. It is strongly recommended to use the individual MRI of the participant to construct the head model, particularly in clinical studies where the source localization is used to guide surgery as for example in epilepsy or in functional mapping of eloquent cortex. If this is not available, a template MRI can be used (for example the MNI brain), but the source localization will be less precise, as shown in Brodbeck et al. (47) in a large patient cohort. The MRI needs several pre-processing steps in order to get to a proper delineation of the gray matter in which the source activity is estimated, and to describe the different compartments of the head (skin, skull, CSF, brain) that have different conductivity parameters. Since the electric field that spreads from the sources to the scalp surface is attenuated by these compartments (particularly by the skull), a proper incorporation of the head shape and the conductivity parameters in the head model is essential for EEG source reconstruction. Once the MRI is pre-processed, the electrodes have to be positioned on the head corresponding to how they were positioned during the recordings. It is obvious that if the position of the electrodes does not correspond to the real position from which the signal was recorded during the experiment, the source localization will not be correct.

### MRI Processing

The head model for EEG source imaging is based on the MRI. As mentioned above, whenever possible the individual MRI should be used. It gives information about the shape of the head, the thickness of the skull and the volume of the gray matter within which the solution points for the source localization are defined. Several processing steps are needed in order to properly extract this information. This includes re-sampling and re-orientation, skull stripping, Bias Field correction and separation of gray and white matter. These processing steps are fairly standard and offered in many different software packages, most well-known in the SPM toolbox (48). While Cartool allows to read MRI images and gray masks that have been processed by other software programs, it also has an integrated MRI processing toolbox. It takes particular care of points that are crucial for a proper layout of the solution points, such as assuring that no holes appear in the gray matter mask and that the sagittal plane is properly determined to assure symmetry of the left and right hemisphere. In the following, the way these processing steps are implemented in Cartool are described:

#### Re-sampling and Re-orientation

Depending on how the MR scanner performed the acquisition and how the participant lied in the scanner, re-sampling and re-orientation of the MRI is needed as a first step. In Cartool, the following geometrical transformations are all built into a 4 × 4 affine transform matrix, which stacks efficiently the successive steps described below, all of them being mathematically linear.

**Figure 2:**
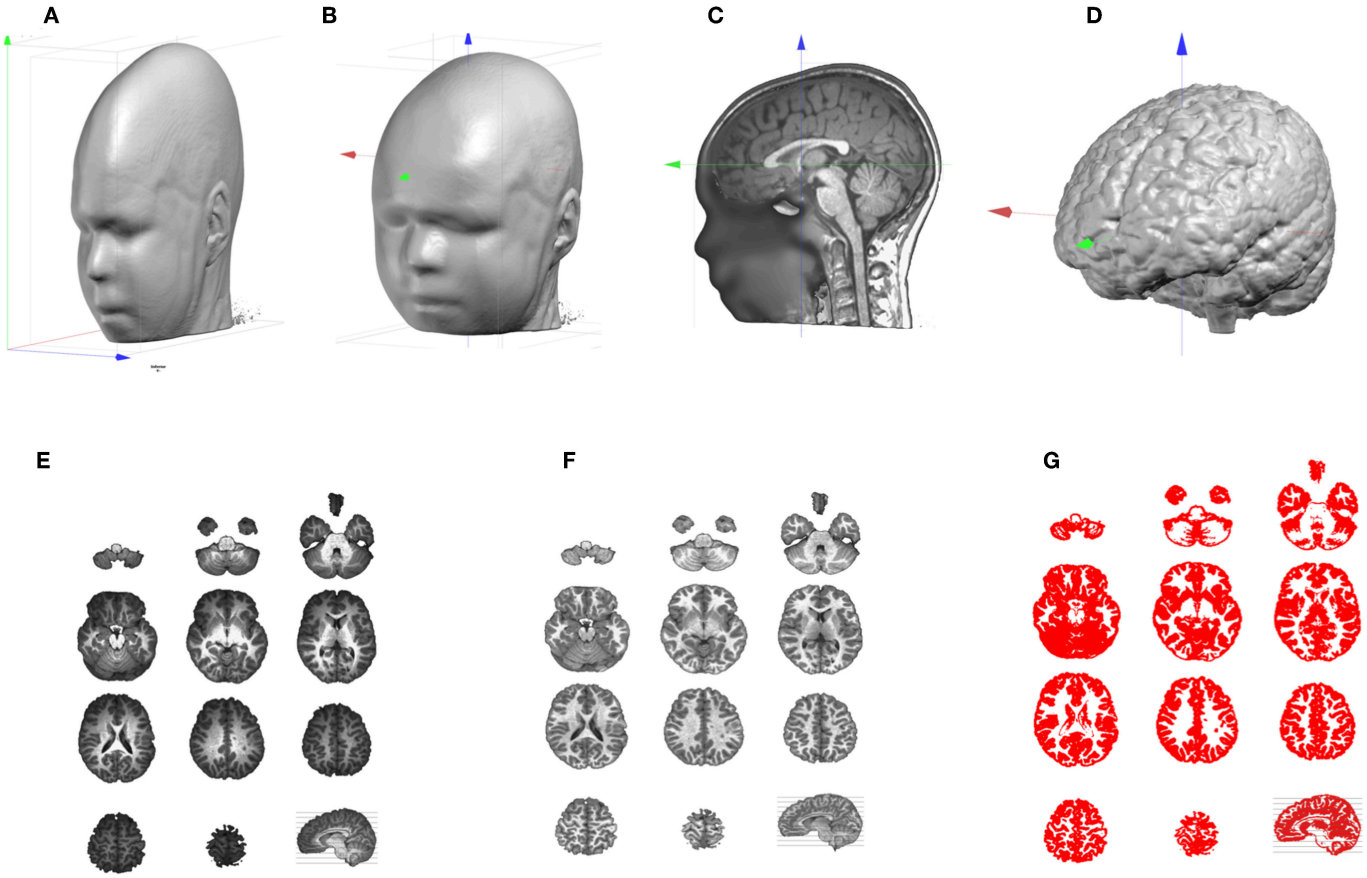
Illustration of the MRI processing pipeline. **(A)** Original anisotropic MRI. **(B)** Result of up-sampling and re-orientation, with red, green, and blue axis pointing, respectively to X, Y, Z. **(C)** Adjustment of the cutting planes and setting of the AC origin. **(D)** Result of the skull-stripping to isolate the brain. **(E)** Brain slices which exhibit the Bias Field of the original MRI. **(F)** Same brain slices post-Bias Field Correction. **(G)** Extraction of the Gray matter.

If the acquisition was anisotropic, as is often the case, voxel sizes are not equal in all three dimensions (Figure 2A). This is very detrimental for any further 3D processing, like filtering, and needs to be addressed as a very first step. In Cartool this is done by simply up-sampling the lowest resolution axes with some linear rescaling, to end up with the highest resolution in all 3 axes. Once the MRI is made isotropic, the axes have to be re-oriented in a standard way to improve readability and compatibility with other software. As a default, Cartool transforms the MRI to the Right-Anterior-Superior (“RAS”) orientation for the three axes X, Y, Z (right-hand system) similar to the MNI template brain. This is done by appropriate 90 degrees rotations (Figure 2B).

Once the main axes have been set, adjustments are performed to further improve readability and comparison across subjects, or comparison with the MNI template. First, an optimal sagittal cutting plane is determined by adjusting 2 rotations values, on the Y and Z axes, and 1 translation value on the X axis, until the two halves defined by this plane are most symmetrical. This is of utter importance for the later stage when laying out the solution points in the brain, because it keeps an anatomically realistic balance between the left and right hemispheres. Once the optimal sagittal plane has been found, the best transverse plane is determined. This is highly recommended as the placement of the participant in the MR scanner varies. A tilted head is normalized in Cartool by adjusting 1 rotation value on the X axis, and 2 translation values in Y and Z. The optimal transverse adjustment is the one that gives a mid-sagittal plane that is most similar to the corresponding mid-sagittal plane of the MNI head. This is done by tilting the head and setting the origin above the anterior commissure. Note that these two steps partly solve the co-registration from a given head to the MNI template (Figure 2C). Only a final rescaling (3 parameters) is needed to achieve the ultimate 9 parameter co-registration. The last geometrical transform is to resample the MRI to reach the desired target voxel size, which is usually 1 mm^3^.

All the steps above are then applied at once on the original MRI, through a 4 × 4 affine transform matrix. Interpolation between the voxels is done with a Lanczos filter, with kernel of size 3, which considers a neighborhood of 216 voxels for each value to be interpolated. The target MRI size is optionally optimized to include only the transformed head, plus some margin, and drop any useless empty spaces.

#### Skull-Stripping and Bias Field Correction

At this point, we should have a standardized individual head. The next step in the pipeline is the skull-stripping to separate skull, CSF and brain (Figure 2D). Two methods are available in Cartool, one mainly based on morphological operators, the other one on region growing. Both methods were designed for T1 MRIs, but appear to be resilient enough to work on T2 or MP-RAGE images.

MRI scans usually have inhomogeneities in space, called Bias Field. Without correcting for it, a given brain tissue like the gray matter will have different values depending on its physical position in the scanner (Figure 2E). This is definitely a non-desired property which will hamper the segmentation of the brain into its constituent tissues. Cartool corrects for the Bias Field of the segmented brain by iteratively equalizing the histogram of the white matter across all 3D directions. Since the white matter has the highest intensity values, it is a good marker for inhomogeneities. Any variations across a given axis are attributed to the Bias Field and are corrected (Figure 2F). By repeating this process across all directions, a global approximation of the Bias Field is determined. The validity of this method is reassured by the final histogram of the brain, which shows very clear-cut tissue separation.

#### Gray Matter Segmentation

The final step of the MRI processing is the separation of white and gray matter. This is needed because EEG source localization usually restricts the source space to the gray matter that contains the synapses where postsynaptic potentials can be generated. Cartool extracts the gray mask by estimating the global intensity distributions of the gray and white matter and the CSF with a Mixture of Gaussians. It classifies each voxel by weighting the Gaussian probabilities, based solely on the voxel intensity, with some neighborhood likelihood (for a given voxel, the greyer the neighbors, the higher the chance to be gray, too). Finally, morphological smoothing operators are applied to fill any possible holes in the gray mask. Note that the produced gray mask is therefore slightly thicker than the actual gray matter, which can be quite thin in some brain areas. The smoothing assures that no gray matter parts are missed (Figure 2G).

### Determining the Solution Points in the Gray Matter

The volume that has been obtained through the gray matter extraction is called the solution space, and constitutes the volume in which the electric activity will be allowed to be localized. The solutions space will typically contain 3000–6000 individual solution points and is thus basically a down-sampled version of the gray mask. Because of the Nyquist theorem, down-sampling should be done with some prior smoothing to prevent aliasing effects. Otherwise, this would result in missing solution points or discontinuities in areas where the spatial frequency is higher than the down-sampled spatial frequency.

In Cartool, finding the optimal solution point distribution is done in the following way, given a number of solution points to attain:

1. An initial down-sampling factor is estimated.
2. The gray mask is down-sampled by the current factor, while remaining centered on the optimal center. This keeps the left and right parts as symmetrical as possible.
3. Solution points with < 8 neighbors out of a neighborhood of 26 are removed, repeatedly for 3 times.
4. The remaining solution points are counted.
5. If the count is close enough to the requested amount of solution points, the process is stopped. Otherwise, the down-sampling factor is up- or down- regulated according to the current numbers, and the process is repeated.

**Figure 3:**
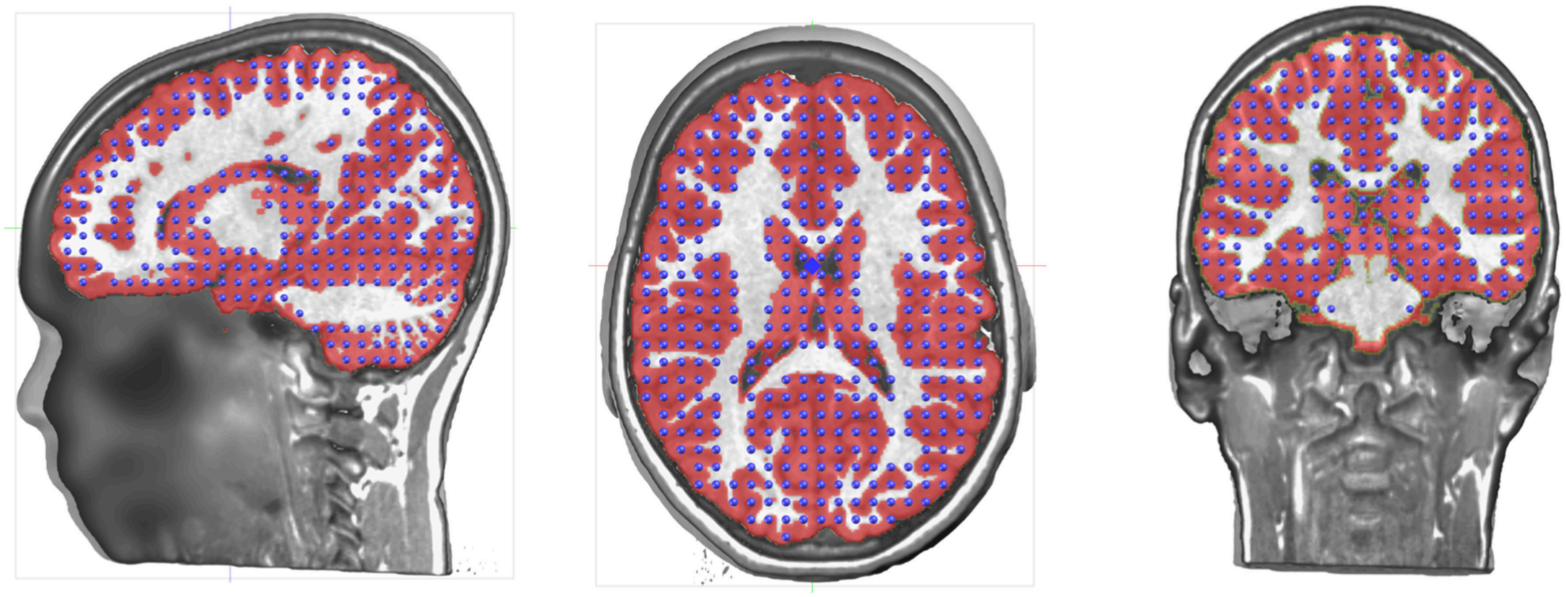
Illustration of the distribution of the solution points in the gray matter.

The solution point extraction is an important step of source localization, very often overlooked, if not totally ignored in the literature. Here are some points that have to be considered:

1. Left-right distribution
  As described above, the MRI has to be realigned to the mid-sagittal plane. That means that the geometrical center of the MRI is going through the YZ plane that cuts the brain in two optimally symmetrical parts. When down-sampling the gray matter into the solution points, the new down-sampled center has to remain in this plane. This will ensure that the resulting solution points will be equally distributed between the left and right hemisphere. Having an asymmetrical distribution of solution points will have an impact on the source localization by giving more weights to one side of the brain and attributing sources to the wrong side. Obviously, a real asymmetrical (pathological) brain will have its mid-sagittal plane set according to its anatomy, and will have an asymmetrical distribution of solution points.
2. Minimum neighborhood
  The inverse process will later need the computation of a discrete Laplacian in the Solution Space. To be able to do that correctly, each solution point has to have enough neighbors. In Cartool, a quite conservative minimum of at least 8 neighbors out of 26 is chosen. Solution points that have less neighbors will be removed.
3. Continuity
  The solution points should cover the whole gray matter without missing points on the thinner parts. It is obvious that source activity cannot be reconstructed on non-existent solution points, leading to a lack of precision for some brain areas. Another risk of missing solution points in some gray matter parts is that neuronal activity coming from this area would be attributed to the solution points closest to the missing part. In order to avoid such effects, Cartool smoothens the gray matter mask as described above to assure continuity of the solution points (Figure 3). This is a desired property and not a defect.
4. Number of Solution Points
  The number of solution points is defined by the user, with a recommended range between 3000 and 6000. There are obvious pros and cons for both low and high number of solution points (Table 2).

**Table 2:** Pros and Cons of the number of solution points in the inverse space.

**Lower number of solution points**	**Higher number of solution points**
(+) Faster to compute the matrices	(–) Longer to compute the matrices
(+) Less memory	(–) More memory
(+) Less numerical precision issues	(–) More numerical precision issues
(+) Smaller matrices and faster display	(–) Larger matrices and slower display
(–) Less spatial resolution	(+) More spatial resolution
(–) Less spatial accuracy	(+) Somewhat more spatial accuracy
(–) Less neighbors around each solution point	(+) More neighbors around each solution point

While computer speed is nowadays only a marginal problem, memory limitations can still be an issue. Numerical precision issues come from the fact that inverting large matrices will cumulate more errors than smaller ones. The spatial resolution (grid spacing) and accuracy (to be spot-on) is a sensitive problem. More points mean more spatial resolution because of smaller grid spacing. This increases accuracy but only up to a limit. Accuracy will stop improving after a given number of solution points (i.e., the inverse solution is not “getting better”) due to the fact that the quantity of information that is put into the system remains the same, and is set by the number of electrodes. Also, the matrix inversion process can intrinsically provide only a limited level of accuracy.

## Number and Positioning of the Electrodes

### Electrode Layout

What is the minimal number of electrodes needed for reliable source localization? This question is often asked, particularly from the clinical community that intends to apply EEG source localization to the EEG that is routinely recorded with the standard 10-20 system, i.e., with only 19 electrodes. Several studies have demonstrated that this low number not only leads to blurring of the solution, but also to incorrect localization (49) compared the effective spatial resolution of different electrode montages (19-129 electrodes) and concluded that “the smallest topographic feature that can be resolved accurately by a 32-channel array is 7 cm in diameter, or about the size of a lobe of the brain”. Simulation studies as well as subsampling studies in epileptic patients with known epileptic focus clearly showed that electrode arrays with < 32 sensors lead to severe mislocalizations and blurring (3, 28). The significant increase in localization precision has been demonstrated by Brodbeck et al. (47) in a large group of epileptic patients where sensitivity and specificity were compared between high-density (128–256 channels) and routine clinical (19–21 channel) EEG. In a cohort of patients with focal ischemic stroke, (50) demonstrated that more than 64 electrodes were needed to avoid mislocalizations of the affected regions. Recent studies showed that the detection and localization of high frequency oscillations, which are potential markers of epileptic areas, are better detected and localized with high- as compared to low-density EEG (51, 52). Also, localization of seizure onset zone using connectivity analysis in the source space was shown to be more precise with high- compared to low-density EEG (53). The fact that the skull resistance is much lower than previously assumed (see section The LSMAC Head Model), additionally supported the notion that high-density EEG is needed to avoid spatial aliasing, that then leads to mislocalization (54, 55). As the skull is much thinner in babies, even more electrodes are needed in this population (56, 57).

Nevertheless, these results do not necessarily mean that imperfect spatial sampling precludes source localization. Even with < 32 electrodes, source localization allows to gain valuable insight about the underlying sources, particularly in applications with well-defined focal activity such as epileptic spikes (15, 58–60).

**Figure 4:**
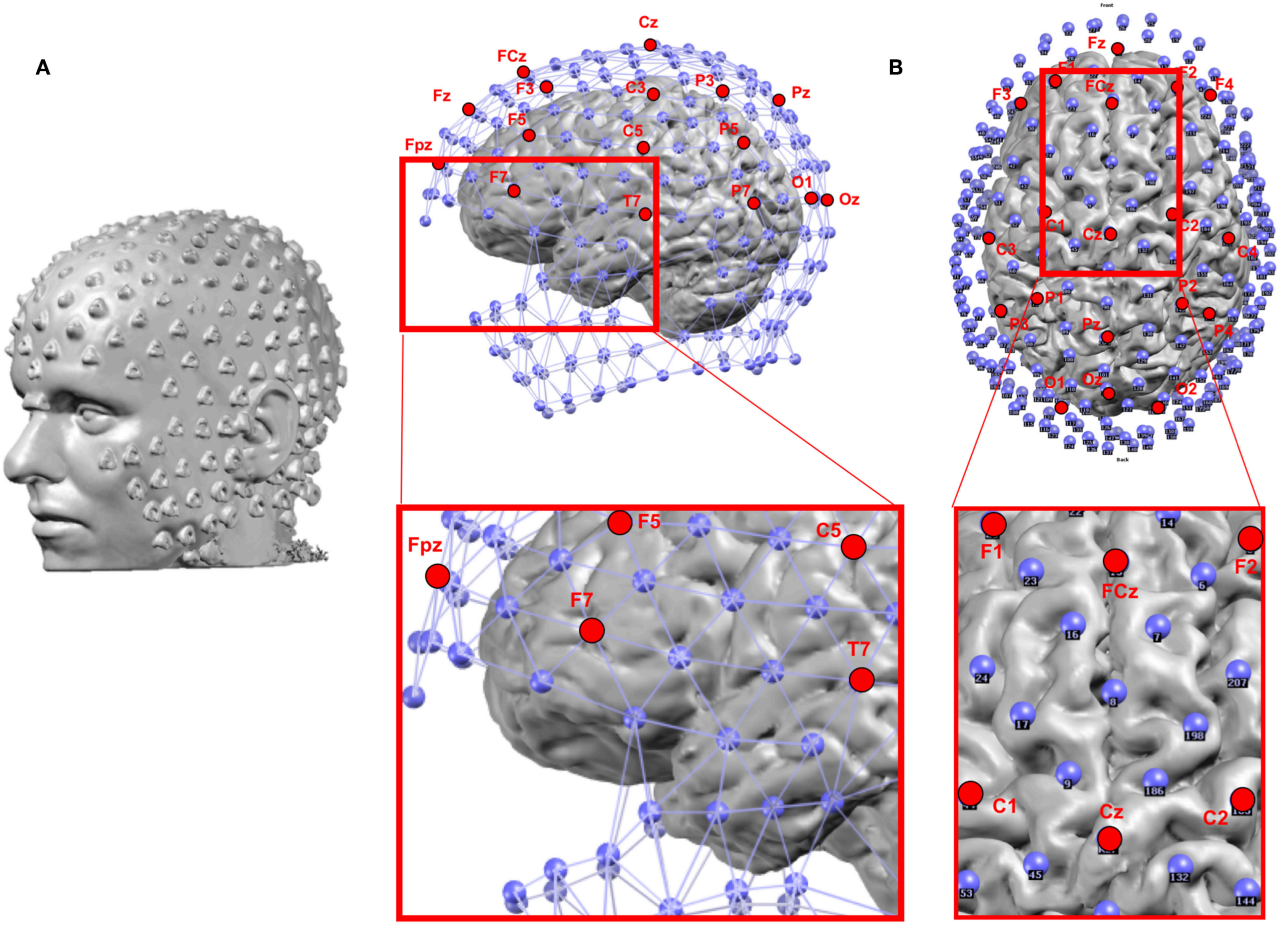
**(A)** Example of the location of 256 electrodes on the head determined by the artifacts that the electrodes create on the MRI image by wearing the EEG net in the scanner. **(B)** Location of the electrodes with respect to the brain: Blue: 256 electrode net. Red: Positions of the 19 electrodes of the standard clinical 10–20 system. The zoomed-in regions show the bad coverage of the frontal, basal temporal and midline areas with the 19 electrodes as compared to the 256 electrodes.

Besides the number of electrodes, their positioning in terms of coverage of the head plays an important role too. The standard 10–20 system does not include electrodes over the inferior part of the head which disfavors the proper recording of activities in the inferior-basal and anterior part of the temporal lobe where activity originating or propagating from the mesial temporal structures is maximal (61, 62) (Figure 4B). Missing these electrodes can lead to mislocalization of activities originating from the mesial temporal lobe (60, 63). It has therefore been recommended that at least 3 inferior electrodes on each side should be added to the standard 10–20 system in clinical routine (64).

### 3D Electrode Positions

The correct positioning of the electrodes on the surface of the head of the participant's MRI is an important point. Ultimately, the position should correspond to the reality, i.e., to the actual position of the electrodes during the recording, as this has a direct impact on the stability of the source localization.

There are different levels of knowledge of the electrode positions during the recordings. Nowadays, EEG caps or nets are usually used, with the advantage of fixed spacing between electrodes. Many studies rely on these fixed positions determined by the manufacturer and the names of the electrode according to the 10-10 coordinate system. A template 3D-array (often provided by the manufacturer) is then used and it is assumed that the EEG cap is placed and adjusted according to some fixed points (Inion, Nasion, preauricular points, Vertex, etc.). It is crucial that this placement is done correctly and it is recommended that photographs are taken to later assure correspondence of the electrodes to these fixed points when landing the electrode array on the MRI head. A more advanced and recommended method, if available, is to measure the actual position of each electrode for each participant using a 3D digitizer or a photogrammetry system (65). The obviously most accurate method is to put the participant in the scanner with the cap on the head and afterwards mark the artifacts induced by the electrodes on the MR images (Figure 4A). This last method bypasses the co-registration procedure described below. However, as it requires an MRI scanner close to the EEG recording room and MRI-compatible EEG caps, this method is rarely possible, except in simultaneous EEG-fMRI studies.

### Co-registration of the Electrodes on the MRI Head

In Cartool, the co-registration of the 3D electrode array is done interactively by displaying and manually adjusting the global 3D shape of the electrode array to the shape of the head. This is a way to make use of all the available geometrical information, instead of relying only on a few fiducial positions. The method can adapt to all cases and allows to co-register either an individual or a template electrode array to either an individual or a template MRI head.

In detail, the following steps are performed interactively: Both the processed (resampled and reoriented) MRI and the electrode array are displayed on the screen. The operator then virtually adjusts the electrode array on the MRI head, mimicking the way the physical electrodes were set on the subject's head. This is done by shifting the electrode positions in any direction, rotating and stretching them until they convincingly look like the reality. Photographs taken during the recording can help to properly adjust the positions.

**Figure 5:**
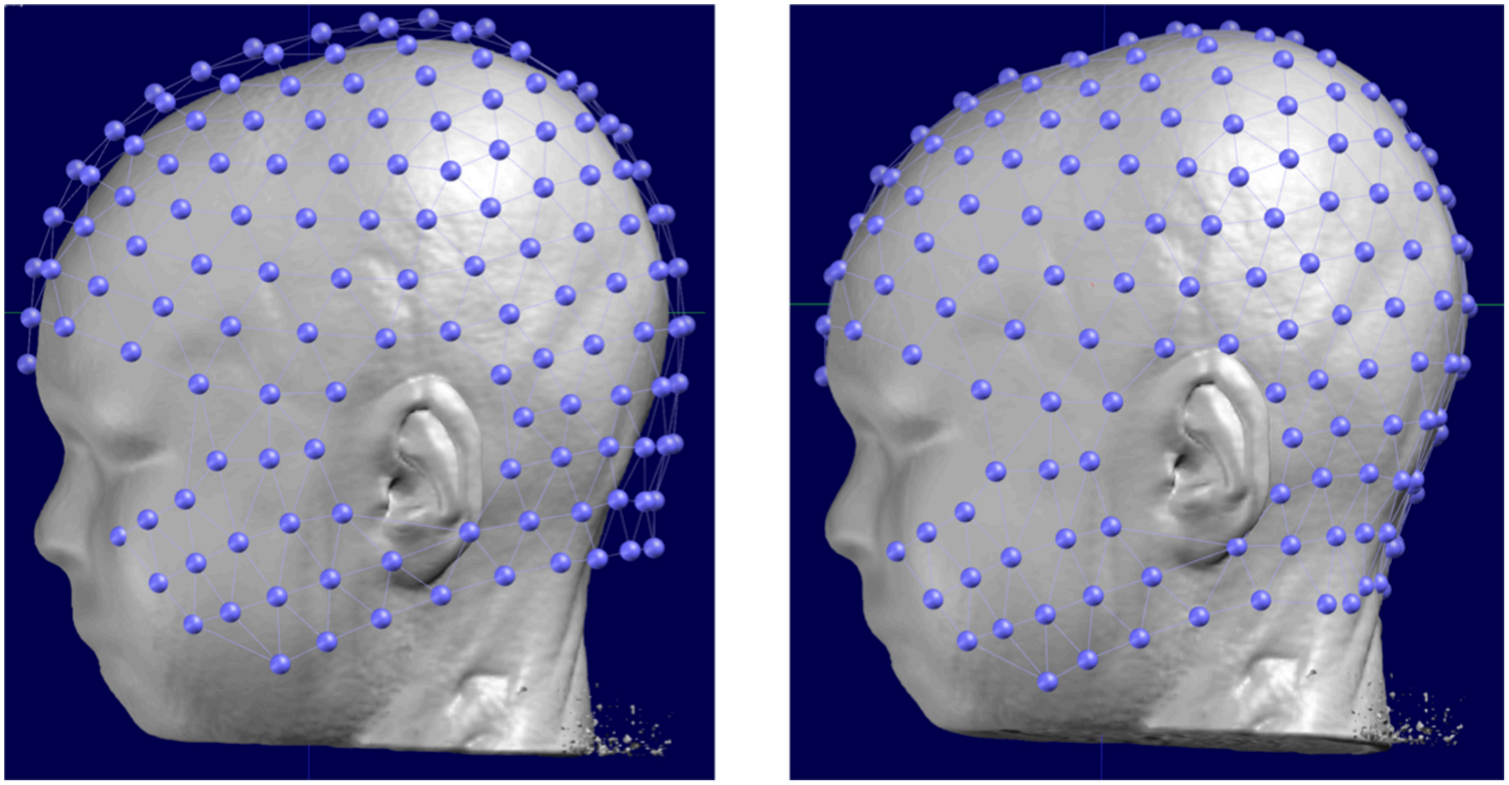
Original position of a template electrode layout with respect to the head of the subject (left) and the corrected positions after manual rotation and translation and the final automatic “gluing” on the scalp.

Once this adjustment is done, Cartool provides a last useful feature: virtually “gluing” the electrodes on the head. For many reasons, like a template electrode array being used on a real MRI head, and no matter how much care is devoted to the previous steps, many electrodes can end up either being below or above the scalp surface. This in turns will be detrimental to the Lead Field computation by biasing the distances from any given electrode to the brain. By activating this virtual gluing, all electrodes will be perfectly projected perpendicularly on the nearest position on the scalp (Figure 5).

## Calculating the Lead Field

In order to calculate the lead field, a head model has to be created that incorporates as realistically as possible the shape of the head and the conductivity parameters of the different tissues between the current sources in the brain and the potential on the scalp. There have been substantial advancements in the construction of realistic head models. Still, even the most sophisticated methods are simplified descriptions of the complex organization of head tissues. The often-used realistic models are the Boundary Element Model (BEM) and the Finite Element Model (FEM). Their superiority compared to 3-shell spherical head models has been demonstrated in simulations (66–68) as well as real data (69, 70). The downside of these sophisticated head models is an increased computational load because numerical solutions have to be applied. They are also more sensitive to any mishap happening during the brain and gray matter extraction, as more brain tissues and more parameters are involved. In Cartool, we implemented a method that we called Locally Spherical Model with Anatomical Constraints (LSMAC, see below). It tries to counteract the computational cost of the BEM and FEM models by using analytical equations while still keeping the realistic aspect of the head geometry and the local variability of the thickness of the skull. Birot et al. (71) compared the LSMAC model with a BEM and a FEM model in a dataset of 38 epileptic patients in whom high-density scalp EEG, intracranial EEG and localization of the resection brain area that rendered the patient seizure-free was available. LSMAC, BEM and FEM were computed from the individual MRI of the patients and source localization was performed on averaged interictal epileptic discharges. Similar source location accuracy with respect to the intracranial recordings and the resected zone was found for all three head models. It was concluded that in such clinical applications, the use of highly sophisticated and difficult to implement head models is not a crucial factor for accurate source localization.

### The LSMAC Head Model

**Figure 6:**
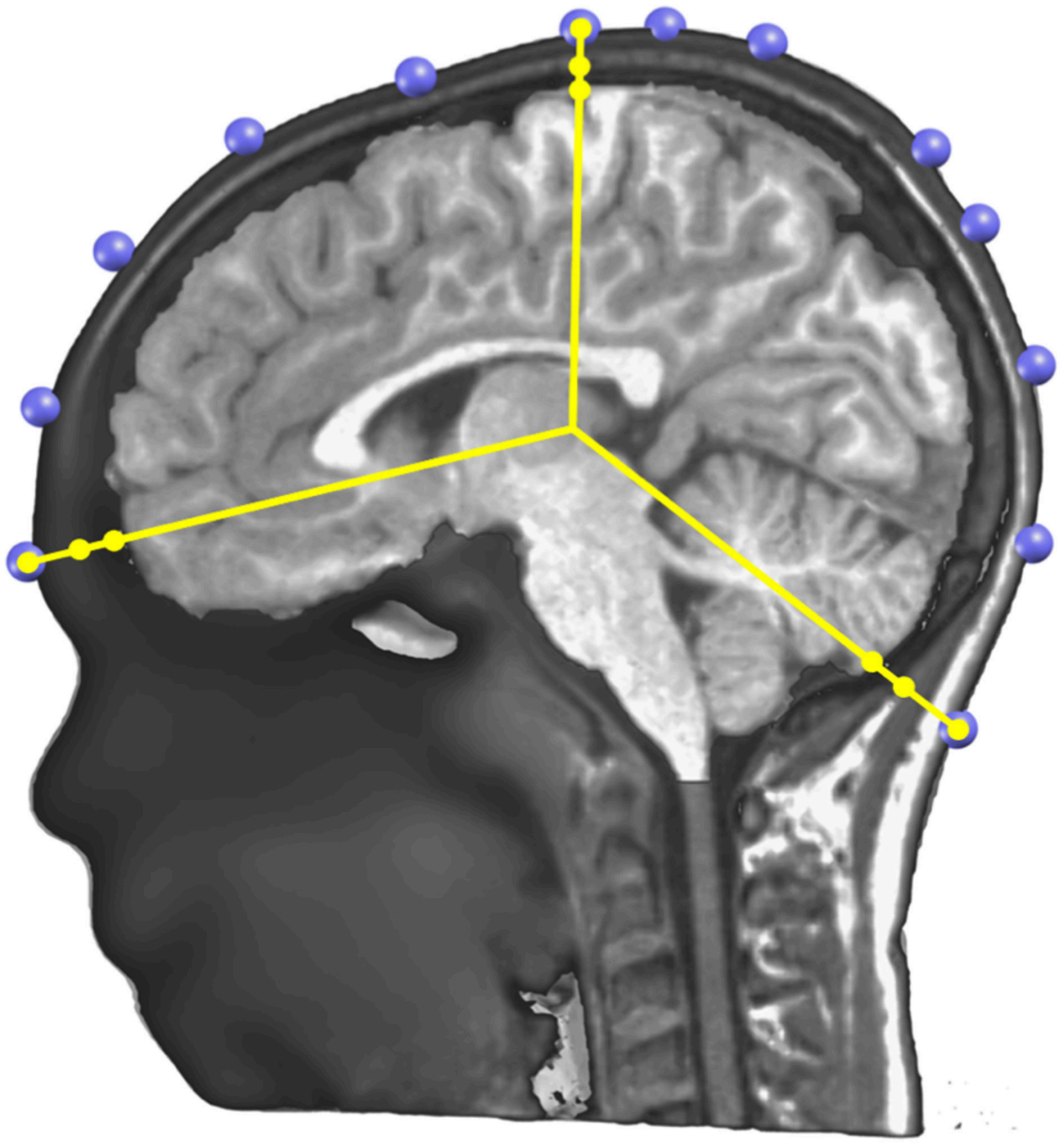
Illustration of the determination of the skull thickness under each electrode. A sagittal cutting plane is shown with the electrodes (in blue) located on the scalp surface, the radius lines (in yellow) extending from the center to each electrode, and on each line three dots showing where the skull and scalp limits are determined.

The Locally Spherical Model with Anatomical Constraints (LSMAC) (29) is an adaptation of the SMAC head model introduced by Spinelli et al. (24). The LSMAC Lead Field calculation requires the pre-processed full head and the gray mask MRIs, the co-registered electrodes and the location of the solution points. Under each electrode, the inner and outer borders of the skull are then automatically determined and the global resistivity value is locally corrected. This decreases the sources of error in EEG inverse modeling. The borders of the skull are determined by analyzing the gray levels of a radial line, going from the center of the brain to the electrode on the scalp. Since the skull is barely visible in T1 MRI scans, it shows up as dark voxels in contrast to the scalp and the brain. Consequently, the beginning and end of the skull can be identified as borders between light and dark voxels on the line. By measuring these borders repeatedly with slight offsets on the scalp, uncertainty pertaining to noise, low voxel intensities and bone structure variability is adjusted. Figure 6 shows an example of the skull radii estimation on 3 electrodes.

**Figure 7:**
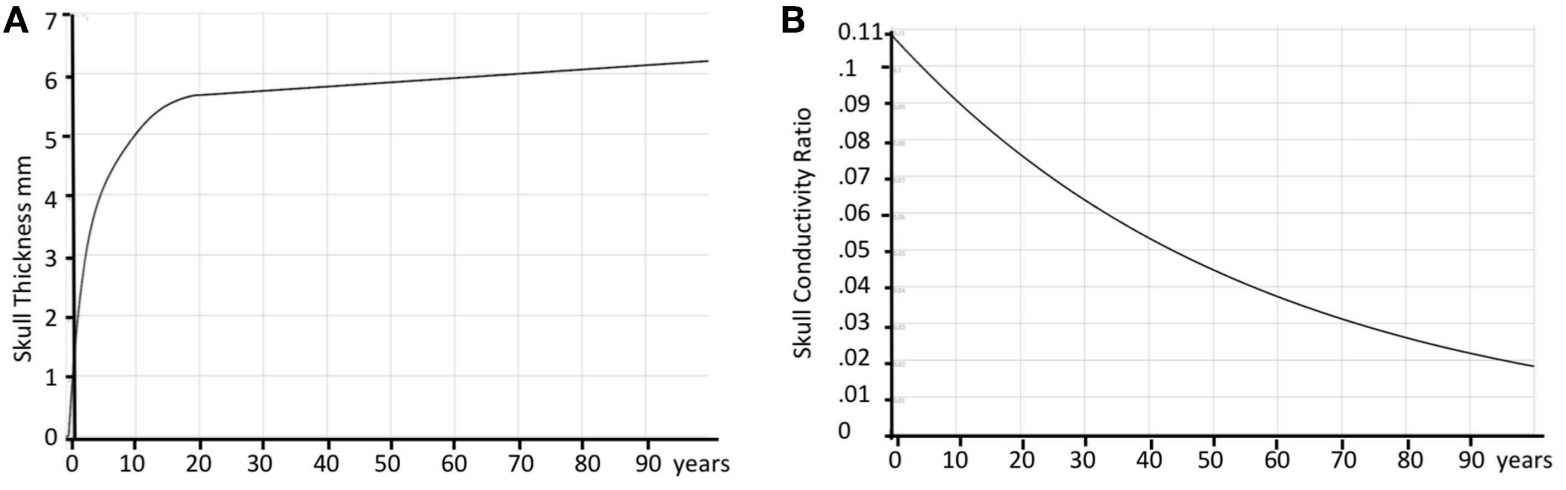
Age correction of skull thickness and skull conductivity. **(A)** Estimated average skull thickness across age. **(B)** Estimated skull conductivity ratios across age.

These skull radii estimate still has some uncertainty due to the nature of the MRI T1 images. To further increase their precision, Cartool requests the user to provide a target age of the subject. Using thickness values described in the literature for different age ranges (72, 73) and linear interpolation of missing values, a curve of the estimated mean thickness for each age was built (Figure 7A). The radii determined from the MRI are then globally rescaled to reach the estimated mean thickness for the given age. This adjustment allows a better estimation of the Lead Field in children, or in the difficult case of newborns. A second advantage of this adjustment is to allow the computation of a Lead Field for any specific age from a fixed template, if the individual MRI was not available.

The skull resistivity has been shown to be much lower than previous literature suggested. The resistivity ratio between the brain and the skull is around 1:10 to 1:30 (74–76), rather than 1:80, as previously assumed (77). Also, the skull resistivity increases with age. Cartool thus has a second built-in curve that gives the relative conductivity of the skull compared to the adjacent tissue (brain, CSF, scalp) as a function of age. The curve is based on a few reported resistivity measures of living tissue (78, 79). According to these reports, the conductivity ratio varied between 1:9.80 (11 years old) to 1:25 (50 years old). An additional estimated value of 1:50 for 100 years old was added to be able to extrapolate the curve past 50 years old, basically following the decreasing trend. When the age of the subject is entered in Cartool, the conductivity value is adapted to this age according to the curve shown in Figure 7B.

## Calculating the Inverse Solution

The inverse problem has no unique solution and a priori assumptions have to be incorporated to derive to a unique assumption of the distribution of neuronal activity in the brain that lead to a certain potential field on the scalp. As explained in the Introduction, a number of solutions of the inverse problem have been proposed, incorporating different constraints based on a priori information about the desired source characteristics or on physiological assumptions [for comprehensive reviews see (3, 25–28)]. In Cartool, we implemented three linear distributed source models: the weighted minimum norm solution (21) the low resolution electromagnetic tomography [LORETA; (22)], and the Local AUtoRegressive Average [LAURA; (23)], all being modifications of the minimum norm (NM) solution (18). We validated these implementations in several experimental and clinical studies by comparing them with intracranial recordings, electrocortical stimulation, fMRI, and clinical outcome after surgery [e.g., (47, 80–84)].

### Regularization Optimization

Tikhonov regularization is typically used in the case of under-determined system of equations, such as when inverting the Lead Field. Simply put, it factors in the equations a level of EEG noise, and enforces a level of smoothness in the inverted results. The more regularization, the smoother the results and the less the sensitivity to noise. However, too much regularization, by over-smoothing the results, will degrade the accuracy of the localization. We wish to use the most precise amount of regularization despite the fact that the amount of noise is not known in advance, and will vary from case to case. To handle all cases, Cartool computes 13 matrices with increasing regularization factors from 0 (none) to 12 (for very noisy data) times a constant ∝, which depends on the selected inverse model. The stack of 13 matrices is then saved into a single file. Later on, when applying an actual EEG to the inverse matrix, its noise level will be evaluated, and the optimal matrix will be chosen.

The general equation for the inverse problem with Tikhonov regularization can be written as:

(2) $$J=W.K^{t}.{\left(K.W.K^{t}+\propto_{R}.I\right)}^{+}.\Phi$$

With *J* being the source density, Φ the electric field, *K* the Lead Field, *W* some specific inverse weighting factors and *I* the identity matrix.

The regularization factor ∝_*R*_ is set the following way, for *R* varying from 0 to 12:

(3) $$\begin{array}{l} \propto_{R}=R.\propto \\ \;\propto =\frac{\max\left(Eigenvalues\left(K.W.K^{t}\right)\right)}{20000} \end{array}$$

The optimal regularization for a given EEG is defined as the L-corner of the norm of the solution points as a function of the regularization factor *R*. When Cartool applies the inverse matrix to the data, it automatically defines this L-corner over the whole dataset and uses this optimal regularization factor for all time points. Alternatively, the user can specify a certain regularization factor for each dataset.

### Normalization of the Inverse Solution Result

When inspecting the estimated current density at each solution point across time in ongoing (non-averaged) EEG it appears that substantial variability of power is observed across solution points. These variations are supposed to come from geometrical and mathematical approximations that are done during the different steps of the inverse matrix calculation. It is thus necessary to find a way to correct for this power variability, in order to reliably estimate the fluctuations of brain activity over time in individual subjects and to compare them between subjects. In Cartool, we implemented a normalization approach by using the background activity of the norm of the inverse solution over time to estimate a baseline and a scaling factor for each solution point. In order to have a robust estimation, a large enough time sample should be used, preferably the whole pre-processed and artifact-excluded data of a given subject. Still, the correction factors can be satisfactorily computed on as little as a thousand time points, as long as no solution point remains in the same stable state more than half of the sampled time, which might be problematic for example in averaged epileptic spikes or in evoked potentials restricted to the time period of sensory processing or motor responses. The normalization should therefore be applied to non-averaged raw data transformed to the source space. A recent study where this normalization method has been used on resting-state EEG to determine the sources of the EEG microstates in task-induced, self-initiated thoughts, showed that this method reveals brain networks that overlap with those derived by fMRI in the same subjects (85).

Here is a step-by-step description of this specialized normalization:

Given a 3D dipole (*sp*_*x*_, *sp*_*y*_, *sp*_*z*_) at a given solution point *sp*, we define *sp*_χ_ as the squared value of its norm:

(4) $$sp_{\chi }=sp_{x}{}^{2}+sp_{y}{}^{2}+sp_{z}{}^{2}$$

The noisy part of the data therefore follows a Chi-square distribution of degree 3.

The variable *sp*_χ_ can be approximated to a normally distributed variable *sp*_*N*_ by (86):

(5) $$sp_{N}=sp_{\chi }{}^{0.2887}$$

Having now a normal distribution, *sp*_*N*_ can be standardized into *sp*_*Z*_ by using the regular z-transform:

(6) $$sp_{Z}=\frac{\left(sp_{N}-\;\mu_{sp_{N}}\right)}{\sigma_{sp_{N}}}$$

However, the values of μ and σ used for the z-transform have to be calculated only on the noisy part of the data—the background activity from the Chi-square i.e., the lowest values of the probability density function. Hence μ is estimated from the left-most Mode of the *sp*_*N*_ distribution:

(7) $$\mu_{sp_{N}}=\;\hat{Mode_{left}}\left(sp_{N}\right)$$

For the same reason, σ is estimated from the Median of Absolute Deviation (MAD), centered on the previously estimated μ, and computed only with the values below μ:

(8) $$\sigma_{sp_{N}}=\hat{MAD_{left}}\left(sp_{N}\right)$$

Both the Mode and the MAD being computed on the left part of the probability density function is key here. In this way it ignores any activity above noise level that might be present in some brain areas while not in others. Rescaling using the actual activities would be incorrect, as it would basically transform them into the baseline. On the other hand, noise can be seen on all solution points and its level is a good estimator of the rescaling that has to be applied. Implementation-wise, these estimators are computed multiple times on random sub-samplings of the data, and the two respective medians of all these estimators are finally taken.

Finally, because we started with positive data (the norm of a dipole), we also wish to end up with positive data in order to avoid any confusion due to having signed results. We define *sp*_*Z*+_ as *sp*_*Z*_ shifted by 3 standard deviations to the right, then divided by 3 so that the background mode is finally aligned to 1.

(9) $$sp_{Z+}=\text{max}\left(\left({\text{sp}}_{\text{Z }}\text{+ 3}\right)\text{/3},\text{ 0}\right)$$

**Figure 8:**
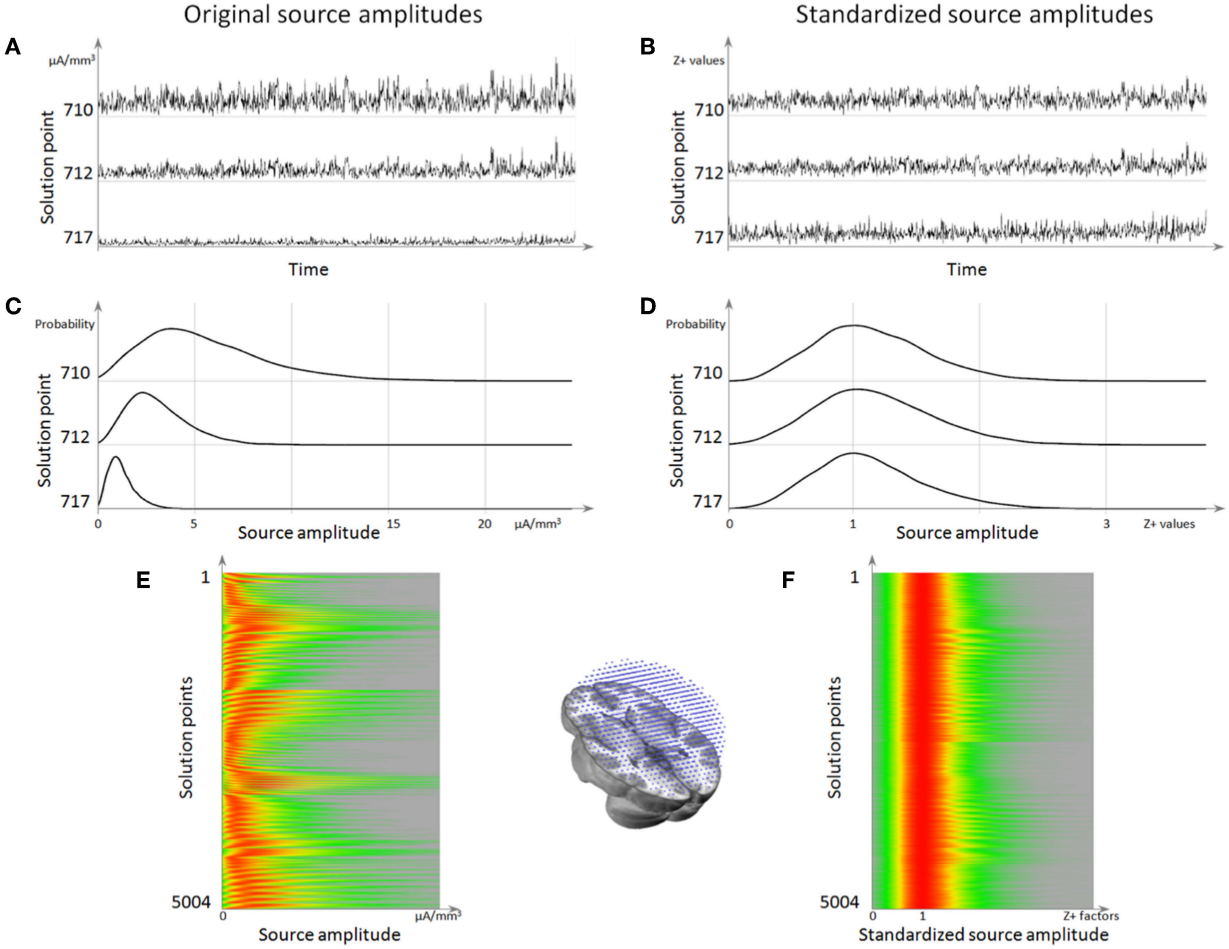
Source localization normalization. **(A)** Time series of 3 solution points showing the difference in mean amplitude (norm) between them. **(B)** The same time series as in **(A)** but after normalization, showing that the 3 solution points now have the same amplitude range. **(C)** Histograms of these 3 solution points, showing that the background activity is the left-most mode of the distribution. **(D)** Histogram after normalization, showing that all the background activity has been centered to 1. **(E)** Histograms for all solution points (vertical axis), with the red color coding for the highest source amplitude probability. **(F)** Histogram of source amplitude probability for each solution point after normalization, showing that all solution points now have a background range from 0 to 1, while retaining their respective highest activities.

After this standardization procedure, the power of the current density is comparable across all solution points, and its noisy component is normally distributed (Figure 8).

### Results of Inverse Solution: Vectorial vs. Scalar

**Figure 9:**
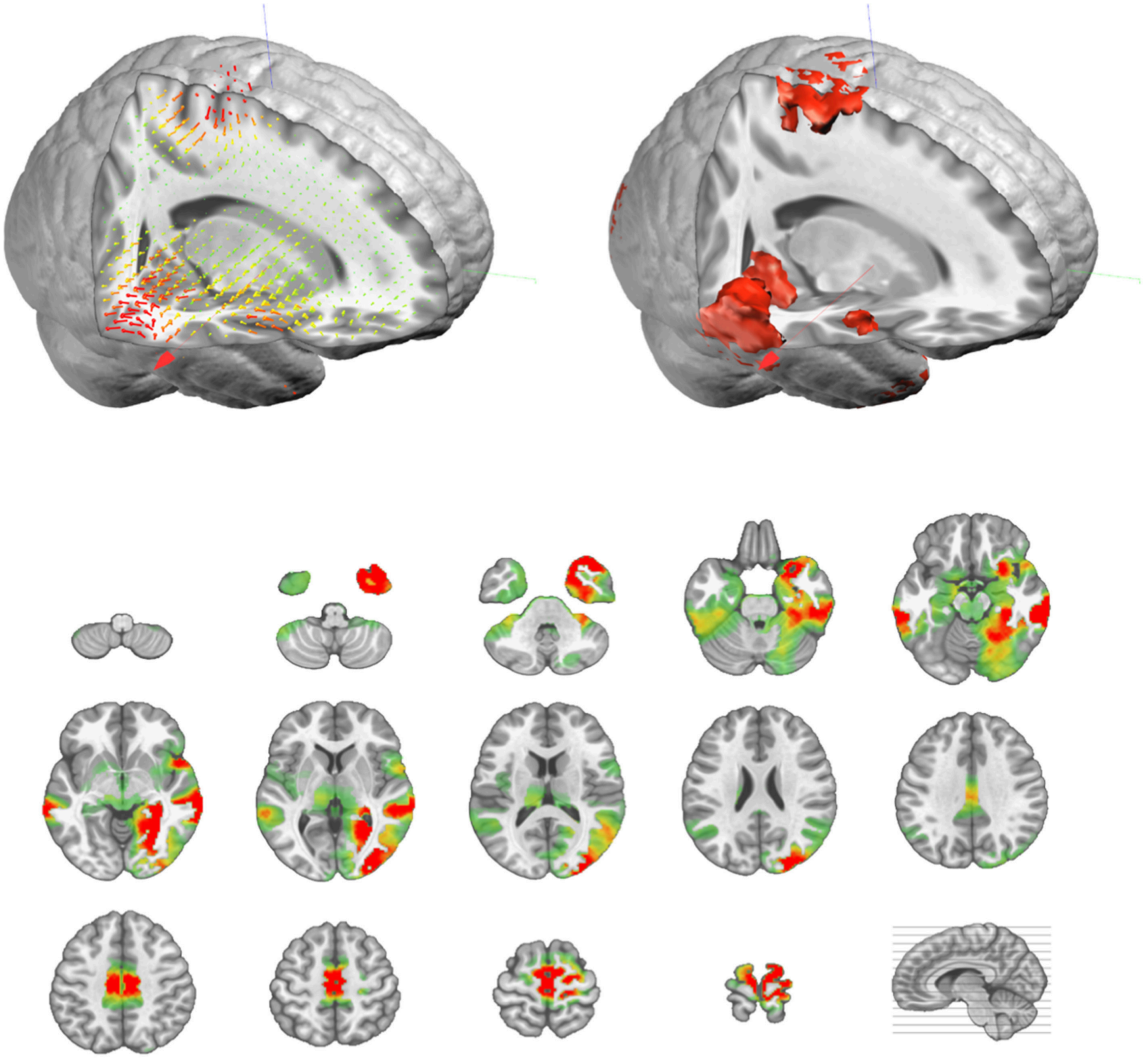
Illustration of the actual vectorial results (top left, in 3D) of the distributed sources, and their corresponding amplitude values (top right, in 3D, and bottom as transverse slices).

The output of the inverse matrix multiplication with the EEG results in equivalent dipoles located on each solution point. As each dipole is a 3D vector, it is described as an amplitude in the x-,y-, and z- directions. In most applications, this vectorial information is not relevant and only the norm (amplitude) of the dipoles is saved, i.e. scalar values. This results in positive values at each solution point as displayed in Figure 9.

## Applications of EEG Source Localization

High-density EEG recordings have become standard in many experimental as well as clinical laboratories, given that most manufactures readily provide such systems and that the application of many electrodes has become fairly easy. It therefore does not come as a surprise that EEG source localization is increasingly used to infer to the areas in the brain that generated the activity observed on the scalp (87–89). Concerning clinical applications, the undoubtedly most intense use of EEG source localization is in epilepsy, with the intention to localize the epileptic zone in pharmaco-resistant focal epilepsies (3, 90). The added value of this method in the pre-surgical assessment of these patients has been demonstrated repeatedly, not only for focus localization, but also for localization of eloquent cortex (3, 47, 83, 91–93). Besides the clinical significance, EEG source localization in epilepsy also gives the unique possibility to evaluate the performance and precision of different head- and source-models because intracranial recordings or the outcome after surgery can serve as “gold-standard” (71, 82, 94, 95). The most direct way to evaluate EEG source localization is the simultaneous recording of scalp- and intracranial EEG. A recent study with high-density (256-channel) scalp EEG recorded simultaneously with intracranial local field potentials from deep brain structures in patients undergoing deep brain stimulation demonstrated that EEG source localization is able to sense and properly localize spontaneous Alpha activity generated in the thalamus or the nucleus accumbens (84). This demonstration opens new doors in the use of high-density EEG source imaging, as it shows that source localization is not restricted to the cortex only.

In experimental studies, EEG source imaging has become standard to localize different brain areas involved in sensory, motor, and cognitive functions, most often applied to event-related potentials (89, 96). However, EEG source imaging is also increasingly used to define large-scale network dynamics by applying connectivity measures (97–99). Because of the high temporal resolution of EEG, functional connectivity measures such as Granger Causality methods are used to study directional connectivity of large-scale networks in the healthy (100–102) and in the pathological (103, 104) brain. It has thereby become clear that such connectivity measures have to be applied in source space and not on the level of the scalp electrodes, since volume conduction and reference-dependency make the interpretability of sensor-based connectivity measures difficult (105–110). Therefore, EEG source imaging is a pre-requisite for functional connectivity analysis [for a recent tutorial paper on EEG connectivity measures see (111)]. It is thus of utmost importance that the source localization is done properly and that the steps described in this review paper are understood and correctly applied.

## Conclusions

**Figure 10:**
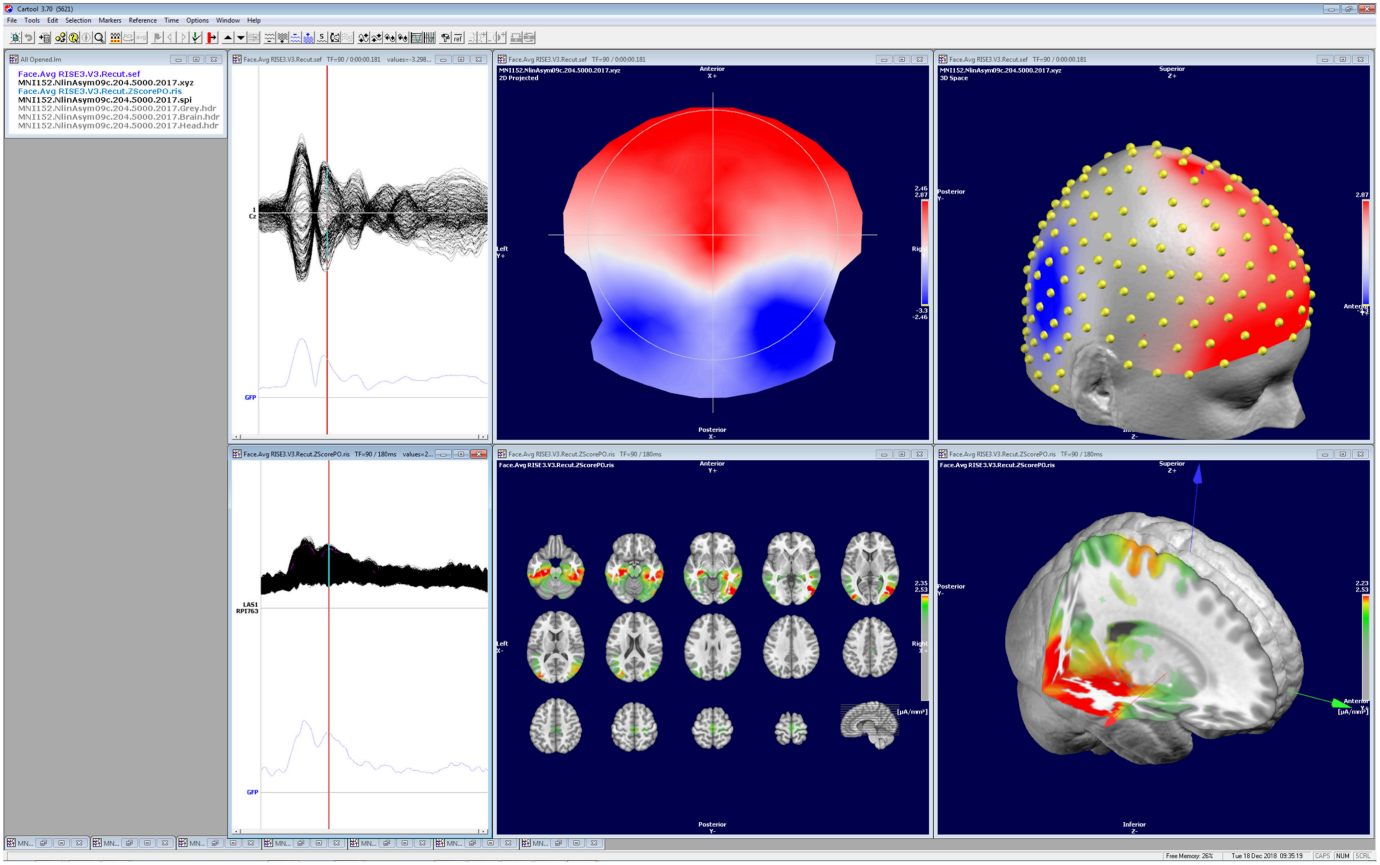
Illustration of the visualization of the data and the results of the different analysis steps as implemented in Cartool. All windows can be independently manipulated in 3D. The screen shot shows a visual evoked potential (face presentation) recorded with 256 electrodes, the corresponding potential map at 188 ms post-stimulus and the estimated sources located in the mesial temporal lobes and the fusiform gyrus.

This review describes in detail the different steps that are needed to derive from a multichannel scalp EEG recording to the estimation of the distribution of the underlying neuronal sources. It explains the logic underlying each step and the requirements that need to be fulfilled to perform them. It illustrates how these steps are implemented in one particular stand-alone software: Cartool. While this might occasionally give the impression of a software manual rather than a review paper, we do not intend to claim that this software is the only one that allows to perform these steps adequately. Several other stand-alone or open-source software packages exist, commercially or freely available, that have implemented these analysis steps in similar or slightly different ways (30); Table 1. We here use the example of Cartool to illustrate the implementation and the usage and to provide a reference for those who use Cartool. In view of the increasing practice of source localization in EEG and MEG applications, it is important that the user well-understands how the software that he/she is using implement the different steps. We also consider it of crucial importance that the data and the results of the analysis are visualized and that the user inspects the data carefully in all different steps and assures that the results make sense (Figure 10).

This review also intends to make the user aware of the obstacles and limitations of each step of the analysis and the choices that have to be made. Basic knowledge of the underlying reasons for these choices and how it is implemented in a given software is mandatory to avoid misinterpretation of the results and to properly describe the methods in a publication. Finally, we hope that this review contributes to the global awareness that EEG source imaging is feasible and doable even for non-engineers and provides information about the function of the human brain that cannot be achieved by analysis restricted to the scalp level.

## Author Contributions

All authors listed have made a substantial, direct and intellectual contribution to the work, and approved it for publication.

### Conflict of Interest Statement

The authors declare that the research was conducted in the absence of any commercial or financial relationships that could be construed as a potential conflict of interest.
